# Supplementary material for: An Exosome-Rich Conditioned Medium from Human Amniotic Membrane Stem Cells Facilitates Wound Healing via Increased Reepithelization, Collagen Synthesis, and Angiogenesis
Source: Cells. 2023 Nov 24;12(23):2698. doi: 10.3390/cells12232698 (PMC10705799; doi:10.3390/cells12232698)
Supplement: Supplementary file 1 [file cells-12-02698-s001.zip › cells-2729232-supplementary.pdf]

cells

MDPI

Supplementary Figure S1

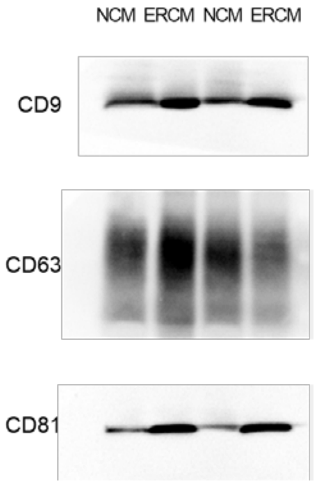

1. Western blot analysis of exosome markers.

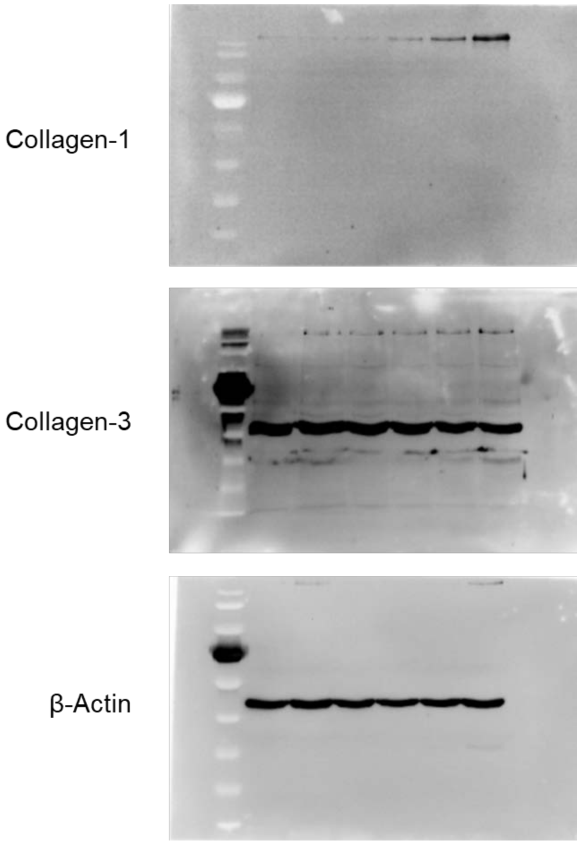

2. Western blot analysis of collagens in vitro.

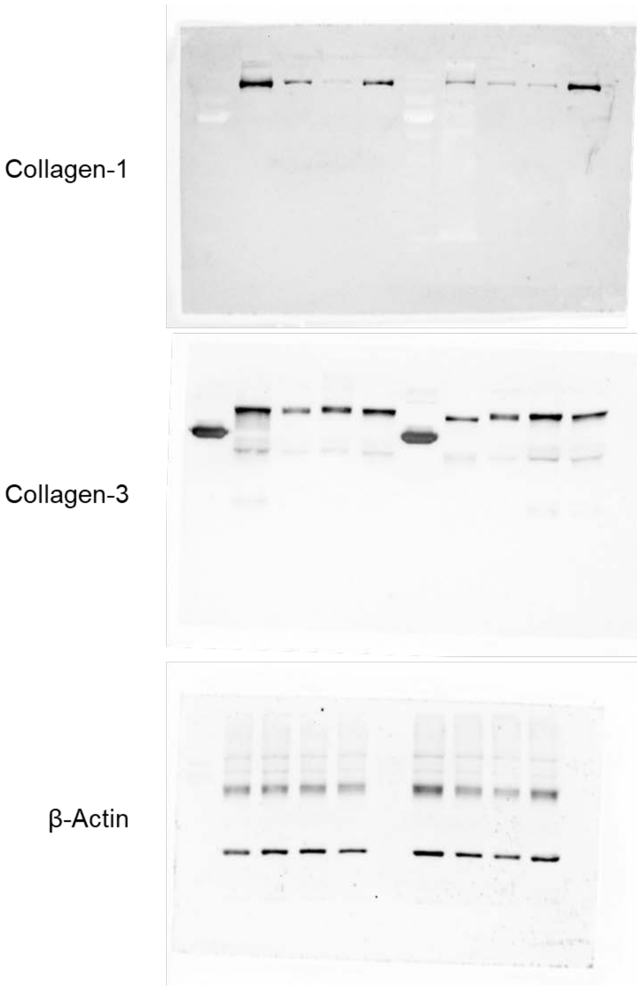

3. Western blot analysis of collagens in vivo.
